# Dynamic Repair Surgery for Late-Stage Facial Paralysis: Advances in Restoring Movement and Function

**DOI:** 10.3390/jcm13164955

**Published:** 2024-08-22

**Authors:** Qing Sun, Xing Li, Zhihui Zhu, Xiting Xiang, Tao Zhang

**Affiliations:** 1 Division of Maxillofacial Surgery, Department of Stomatology, Peking Union Medical College Hospital & Chinese Academy of Medical Science, Beijing 100730, China; sunqing9701@student.pumc.edu.cn (Q.S.); lixing@student.pumc.edu.cn (X.L.); zhuzhihui@pumch.cn (Z.Z.); s2023001055@pumc.edu.cn (X.X.); 2 Department of Plastic and Reconstructive Surgery, Peking Union Medical College Hospital & Chinese Academy of Medical Science, Beijing 100730, China

**Keywords:** late-stage facial paralysis, dynamic repair, nerve and muscle transplantation, muscle flap, tendon transposition

## Abstract

**Purpose:** Facial paralysis results from congenital or acquired facial nerve damage, leading to significant cosmetic and functional deficits. Surgical resection of parotid and midface tumors can cause facial paralysis, necessitating effective treatment strategies. This review addresses the challenge of restoring movement and function in late-stage facial paralysis, focusing on dynamic repair techniques involving nerve and muscle transplantation. **Methods:** The review encompasses studies on dynamic repair surgery for late facial paralysis, including techniques such as local muscle flap with pedicle transfer, vascularized nerve flap with pedicle transfer, and multiple muscle flap procedures. A systematic literature search was conducted using PubMed, Web of Science, and Google Scholar, covering studies from 2000 to 2024. Keywords included “dynamic repair”, “late-stage facial paralysis”, “nerve and muscle transplantation”, “muscle flap”, and “tendon transposition”. Included were clinical studies, systematic reviews, and meta-analyses reporting surgical outcomes. Exclusion criteria included studies with insufficient data and non-peer-reviewed articles. **Results:** Dynamic repair techniques involving nerve and muscle transplantation are essential for treating late-stage facial paralysis. Each surgical method has strengths and limitations. The masseter muscle flap demonstrates high success rates, although it can cause horizontal tension and jaw contour issues. The temporalis muscle flap is effective for smile restoration but may lead to temporal concavity. The gracilis muscle flap is widely used, especially with dual nerve innervation, showing high success in spontaneous smiles but requiring a longer recovery period. The latissimus dorsi flap is effective but can cause edema and shoulder issues. The serratus anterior free flap offers flexibility with precise vector positioning but may not achieve adequate lip elevation and can cause cheek swelling. Combined multi-flap surgeries provide more natural facial expressions but increase surgical complexity and require advanced microsurgical skills. **Conclusions:** Dual nerve innervation shows promise for restoring spontaneous smiles. One-stage surgery offers faster recovery and reduced financial burden. Comprehensive patient evaluation is crucial to select the most suitable surgical method. Dynamic repair techniques involving nerve and muscle transplantation provide effective solutions for restoring function and aesthetics in late-stage facial paralysis. Future research should focus on long-term outcomes, patient satisfaction, and standardizing surgical protocols to optimize treatment strategies.

## 1. Introduction

Facial paralysis is characterized by the reduction or complete paralysis of facial muscles due to congenital or acquired facial nerve damage. In the field of head, neck, and maxillofacial surgery, the resection of parotid and midface tumors can lead to partial or complete facial paralysis with complex soft tissue defects. In 2021, a study on primary parotid gland carcinoma revealed that facial paralysis was observed in 6.7% of patients preoperatively. Among them, 11.7% of the patients underwent radical resection of the parotid gland. Notably, 5% of the patients had normal preoperative facial function, but due to the intraoperative detection of tumor infiltration into the facial nerve, radical surgery was required, resulting in immediate postoperative facial paralysis in 34.4% of the patients [1]. Umberto and colleagues conducted a retrospective study of 554 patients with benign parotid gland tumors who underwent parotid gland surgery from 2012 to 2021. They analyzed the incidence of complications between extracapsular dissection (ECD) and superficial parotidectomy (SP). There were 16 cases (4.49%) of temporary facial paralysis in ECD patients and 35 cases (17.67%) in SP patients. There were 8 cases (2.25%) of permanent facial paralysis in ECD patients and 13 cases (6.56%) in SP patients [2]. Prolonged denervation of the facial muscles can result in irreversible atrophy and fibrofatty degeneration. In most cases, these pathological changes occur within one and a half to two years after the onset of paralysis [3]. Patients with facial paralysis lasting more than two years find it difficult to restore nerve function through surgery and are referred to as late-stage facial paralysis.

In cases of complete facial paralysis, the loss of resting tone in the upper lip retractors (such as the zygomaticus minor, levator labii superioris, and levator labii superioris alaeque nasi) results in the disappearance of the nasolabial fold and displacement of the upper lip towards the unaffected side. The loss of resting tone in the buccinator, zygomaticus major, and levator anguli oris muscles causes drooping of the mouth corner. Treating late-stage facial paralysis is highly challenging, with the ultimate goal of re-establishing facial symmetry and muscle function to achieve maximum static and dynamic harmony. This requires dynamic repair surgery techniques involving nerve and muscle transplantation to improve facial muscle function. Surgical reconstruction should target the affected areas: the lips, mouth corner, midface, eye region, or forehead. Timely multidisciplinary treatment can significantly enhance the patient’s quality of life [4]. This review addresses the challenge of restoring movement and function in late-stage facial paralysis, focusing on dynamic repair techniques involving nerve and muscle transplantation.

## 2. Materials and Methods

The review encompasses studies on dynamic repair surgery for late facial paralysis, including techniques such as local muscle flap with pedicle transfer, vascularized nerve flap with pedicle transfer, and multiple muscle flap procedures. A systematic literature search was conducted using PubMed, Web of Science, and Google Scholar. Keywords included “dynamic repair”, “late-stage facial paralysis”, “nerve and muscle transplantation”, “muscle flap”, and “tendon transposition”. Studies from 2000 to 2024 were included. Inclusion criteria were clinical studies, systematic reviews, and meta-analyses with reported surgical outcomes. Exclusion criteria included studies with insufficient data and non-peer-reviewed articles. Ethical exemption was granted due to the review nature of the study, with no direct involvement of human or animal subjects.

### 2.1. Pedicled Local Muscle Flap and Tendon Transfer

#### 2.1.1. Masseter Muscle Flap

For local flaps, masseter and temporalis muscle transposition techniques can be selected to restore a paralyzed smile. Their blood supply and nerve innervation allow for appropriate adjacent transfer without significant risks of necrosis or denervation [5]. However, utilizing the masseter muscle as an independent muscle transfer can result in undesired horizontal tension and lack of a proper vector to elevate the upper lip, potentially exacerbating corner-of-the-mouth deviations [5]. In a 2012 study, physicians Damir B et al. performed [6] pedicled masseter muscle transfer (PMMT) on three patients in a study. In the clinical cases, the mean commissure movements of the paralyzed and normal sides were 7 mm and 12 mm, respectively. The mean angles of commissural movement for the paralyzed and normal sides were 62° and 59°, respectively. Their observations suggest that the PMMT provided less excursion of the commissure upon smiling compared to free gracilis muscle transfers. Donor-site hollowing needs to be addressed by the use of either local flaps, free dermal fat grafts, and/or secondarily by fat grafting. Additionally, the deep anatomical position of the masseter muscle poses surgical challenges and postoperative complications, such as lower jaw contour defects, which are not uncommon, limiting its clinical application [7]. In practice, the masseter muscle can be utilized as an adjunct muscle transfer to supplement the temporalis muscle.

#### 2.1.2. Temporalis Muscle Flap

The temporalis muscle flap, innervated by the deep temporal branch of the trigeminal nerve, is widely employed in the treatment of facial paralysis due to its proximity to the surgical site and the absence of the need for nerve coaptation [8]. In 1934, Dr. Gillies introduced the temporalis muscle flap transposition technique, which involves detaching the temporalis muscle from its temporal fossa attachment, rotating it over the zygomatic arch, and securing it to the orbicularis oris muscle [8]. A significant drawback of this technique is the potential for temporal hollowing and bulging at the zygomatic arch, resulting in poor aesthetics [9]. In 1953, Dr. McLaughlin described a method of temporalis tendon advancement, which involves severing the coronoid process of the mandible to reposition the temporalis tendon, extending the fascia to secure it to the mouth corner [10]. In 1997, Dr. Labbé [11] further developed this into lengthening temporalis myoplasty (LTM), which accesses the coronoid process through the temporal fossa (superior approach), achieving greater anatomical exposure of the superficial temporalis plane by sectioning the zygomatic arch. An alternative inferior approach through the nasolabial fold allows coronoid process sectioning while preserving the zygomatic arch [12]. By preserving the superficial temporal fat pad and dissecting above the deep temporal fascia, temporal concavity can be effectively avoided [13,14]. Subsequently, scholars have proposed an endoscopic intraoral approach to minimize facial skin trauma [15]. Cruise et al. argued that the temporalis muscle flap can improve lip control and chewing efficiency while restoring symmetry to the perioral region [16]. In 2020, Dr. Wang and colleagues [17] reported on a partial temporalis muscle flap transposition technique, with an anterior incision 2 cm above the zygomatic arch and a posterior incision extending downward to the preauricular area. The rotation of the temporalis muscle flap aligns with the direction of the smile. Postoperative results showed improvements in both static and dynamic scores, with incisions hidden within the hairline and aesthetically pleasing outcomes.

However, as the temporalis muscle is innervated by the deep temporal nerve of the trigeminal nerve, chewing-induced muscle contractions can cause eye closure, necessitating prolonged dynamic training and potentially multiple adjustments to tendon length and tension [17].

### 2.2. Blood Vessel and Nerve-Based Free Muscle Flap Transplantation for Reconstruction

#### 2.2.1. Gracilis Muscle Flap

The gracilis muscle is commonly used as a donor muscle for free muscle transfer, primarily nourished by branches of the deep femoral artery and accompanied by two veins, with nerve supply from the anterior branch of the obturator nerve [18]. This muscle offers advantages such as ease of harvesting, minimal functional impact on the donor site, consistent neurovascular anatomy, and segmental distribution of nerves and vessels [19]. In 1976, Dr. Harii [20] first reported the use of gracilis muscle free transfer (GMFT) with a neurovascular pedicle for the treatment of late-stage facial paralysis, innervated by the ipsilateral masseter nerve (MN). Subsequent reports by Brien et al. [21] and Vedung et al. [22] described the use of cross-facial nerve graft (CFNG) with the contralateral FN as the motor source for gracilis muscle transfer. This technique typically involves two stages: the first stage creates the CFNG. After 9 to 12 months, when the regenerating axons from the nerve graft reach the paralyzed-side earlobe, the second surgery is performed when Tinel’s sign is positive [23]. This involves transferring the free gracilis muscle flap and performing a secondary anastomosis with the distal end of the sural nerve. Thus, the contralateral facial nerve (FN) becomes the preferred motor source for restoring natural facial expressions in free muscle flap transplantation.

**Figure 1:**
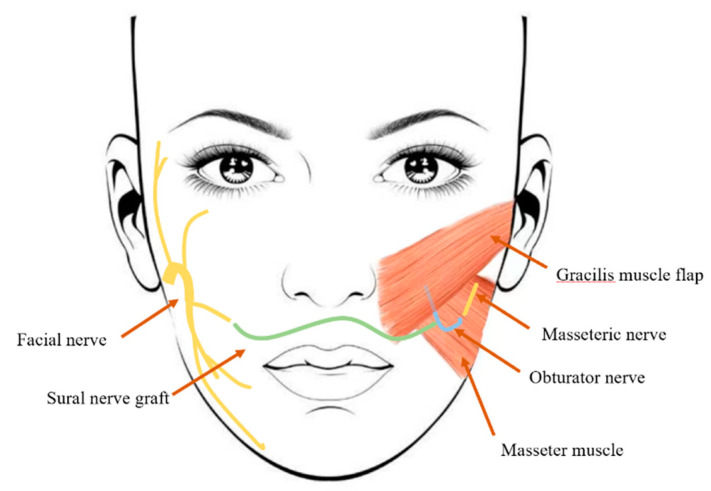
Schematic representation of the transfer of the gracilis muscle flap to the face. The flap is innervated by the masseteric nerve (end-to-end anastomosis) and the contralateral facial nerve (end-to-side anastomosis) via an interpositional sural nerve graft.

In 2012, Biglioli [24] reported a one-stage regeneration procedure utilizing dual nerve innervation (DI). They used end-to-end suturing to connect the anterior division of the obturator nerve with the ipsilateral masseteric nerve and the sural nerve with the contralateral facial nerve (Figure 1). This method combines FN stimulation to ensure spontaneous movement with MN input to ensure contraction strength. In 2015, Cardenas-Mejia et al. [25] performed end-to-end suturing between the anterior division of the obturator nerve and the contralateral facial nerve, as well as between the masseteric nerve and the contralateral facial nerve. In 2019, Alessio Baccarani and colleagues [26] proposed a method in which the obturator nerve was longitudinally split into two separate free nerve grafts, each being end-to-end sutured to the ipsilateral masseteric nerve and the sural nerve. A challenge with this approach is that the decision to proceed with the surgery depends on the intraoperative assessment of the diameter of the obturator nerve using a microscope. An alternative approach described by Uehara et al. [27] involves suturing the masseteric nerve to the intramuscular nerve branches of the gracilis muscle while connecting the obturator nerve directly to the cross-facial nerve graft. Dusseldorp et al. [28] evaluated the ability of dual nerve-innervated gracilis muscle flaps to produce spontaneous smiling and found that 89% of patients with functionally innervated dual nerve flaps achieved different degrees of spontaneous smiling. The dual innervation technique for free gracilis muscle transfer is considered safe, with activation times similar to those of single innervation using only the MN [29]. Michael et al. [30] suggested that dual innervation is particularly beneficial for elderly patients who require enhanced axonal input. In a meta-analysis published in 2023, one hundred and forty-seven articles containing FGMT were systematically reviewed. Thirteen studies, encompassing 435 observations (179 CFNG, 182 MN, 74 DI), were eligible for meta-analysis. The mean change in commissure excursion was 7.15 mm for CFNG, 8.46 mm for MNM, and 5.18 mm for DI [31].

Due to continuous exploration and refinement by clinicians, gracilis muscle flap transfer has become the most widely used method for treating facial paralysis, offering both aesthetic and functional benefits.

#### 2.2.2. Latissimus Dorsi Flap

**Figure 2:**
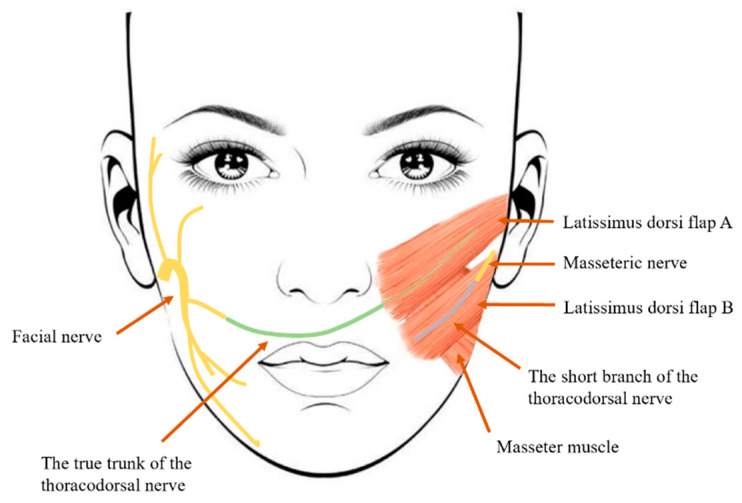
Schematic representation of the transfer of the latissimus dorsi muscle flap to the face. Muscle A, carrying the true trunk of the thoracodorsal nerve, is innervated by the contralateral facial nerve and proximally fixed to the nasolabial region. Muscle B, carrying the short branch of the thoracodorsal nerve, is innervated by the masseteric nerve and distally fixed to both the nasolabial and angular regions of the mouth.

The latissimus dorsi (LD) flap is a commonly used transplant area in microsurgery, supplied by the thoracodorsal artery and secondary intercostal vessels. It has a stable and rich blood supply with a concealed donor site [32]. In 1988, Harii et al. [33] first proposed a one-stage surgical approach for facial paralysis treatment involving the LD flap, which is innervated by the thoracodorsal nerve (TDN) with a length of 15 cm, and anastomosis with branches of the contralateral facial nerve. This method effectively restores spontaneous and natural smiles, achieving good synchronization between the paralyzed and non-paralyzed sides [34]. However, the strength of the muscle contraction can be inconsistent, likely due to the inevitable axonal loss when preparing a 15 cm length of the TDN by severing its side branches. In 2009, Watanabe et al. [35] described a dual innervation technique, where the LD flap is reinnervated by anastomosing it to both the contralateral FN buccal branch and the ipsilateral masseter motor nerve, thus restoring synchronized spontaneous smiles on both sides of the face. The drawback of this method is that the thick flap can lead to facial bulkiness. Dr. Takushima et al. [36] noted that single-stage LD transfer sometimes results in muscle weakness, especially in middle-aged male patients with substantial cheek fat. For these patients, using the ipsilateral trigeminal nerve might be preferable to the contralateral FN branch. In 2015, Okazaki et al. [37] reported a method using separated LD flaps for single-stage multi-flap transfer, where one flap’s TDN is anastomosed to the contralateral FN, while the short branch of the TDN innervating another flap is sutured to the ipsilateral MN (Figure 2). This method avoids the uncertainty of dual nerve innervation and the need for nerve transplantation alternatives. However, separating the flaps based on neurovascular anatomy can be challenging, and involuntary movements might occur due to MN stimulation during chewing. In 2022, Park et al. [38] evaluated the efficacy of single-sided (ipsilateral masseteric nerve only) or double-sided (ipsilateral masseteric nerve plus contralateral buccal branch of the facial nerve) innervation of the LD flap for facial paralysis reconstruction. They found that partial LD transfer with dual innervation produced more positive outcomes for spontaneous smiles. In 2023, Dr. Lee et al. achieved similar results, showing significant improvement in dynamic smile symmetry with dual innervation, with no difference in resting state improvements between groups.

The disadvantages of the LD flap include the need for lateral decubitus positioning during surgery, preventing simultaneous operation by two surgical teams, and the muscle’s thickness, which can cause facial bulkiness. Additionally, there may be shoulder and back pain, affecting shoulder mobility.

#### 2.2.3. Serratus Anterior Free Flap

The serratus anterior free flap (SAFF) is characterized by a long neurovascular pedicle consisting of the long thoracic nerve (LTN) and the serrated branches of the thoracodorsal vessels. This flap offers flexibility in skin paddle selection and can carry multiple muscle bundles simultaneously [39]. Due to its thin, fan-like appearance, the SAFF is well-suited for dynamic facial reanimation. In 1982, Dr. Buncke and colleagues first proposed using the SAFF for facial paralysis reconstruction. Dr. Yoleri [40] noted that the SAFF allows for adjustable tension and precise vector positioning of the serratus anterior muscle bundles, resulting in stronger open-mouth smiles and more natural three-dimensional lip closure smiles.

Subsequent exploration has shown that, in most cases, two muscle bundles are used to restore the function of the zygomaticus major and the levator labii superioris, which are responsible for midface and lower face movements [41]. The scapular origin (proximal one-third) of the muscle bundles is positioned at the mouth corner, fixed to the orbicularis oris (zygomaticus major) and the alar base (levator labii superioris). Both sides can be trimmed and secured to the deep temporal fascia and preauricular fascia. In 2019, Dr. Hisashi Sakuma and colleagues [42] reported a method involving the transplantation of two to three superficial serratus anterior muscle bundles, reinnervated by the MN, to the upper lip, lower lip, and mouth corner at different vectors. However, due to the close proximity of the perioral muscles, achieving sufficient vector differentiation can be challenging, leading to inadequate lip elevation or cheek bulging during muscle contraction. In 2021, the same team reported [43] a dual-nerve innervation, multi-vector transfer technique for the superficial serratus anterior muscle, fixing two muscle bundles to the upper lip and mouth corner, then using a horizontal broad fascia graft to pull the lower lip outward. The upper muscle bundles primarily aim to elevate the upper lip, producing a more natural smile. Spontaneous contractions were observed 5–6 months post-surgery [44,45]. This is much faster than the typically observed spontaneous contraction at 9–12 months after cross-facial nerve grafting. Studies have shown that the dual-nerve innervated SAFF significantly outperforms the MN-innervated SAFF in terms of lip symmetry and resting tension restoration, ensuring symmetry in both resting and voluntary smiles [46]. However, further improvement in surgical techniques may be needed to achieve better aesthetic outcomes for spontaneous smiling.

A systematic review in 2023 [47] indicated that 81.3% of SAFF cases involved single-stage soft tissue reconstruction and dynamic facial restoration. Given that most elderly patients require adjuvant therapy following radical malignant tumor resection and have limited life expectancy, single-stage surgery presents a significant advantage [48,49]. Additionally, there are few reported donor site complications associated with the SAFF [50].

#### 2.2.4. Others 

Besides the three mentioned flaps, other donor muscles used include the rectus abdominis, pectoralis minor, and sternohyoid muscles. Due to limited literature, a brief overview is provided below.

In 2006, Dr. Sajjadian and colleagues [51] introduced a single-stage neurovascular free flap reconstruction using the rectus abdominis muscle and accompanying intercostal nerves in five patients with unilateral facial paralysis. Although postoperative symmetry and voluntary movement improved, they remained below normal levels. This represented a significant step forward in the search for functional dynamic facial reconstruction techniques. In 2012, Harrison and colleagues [52]. reported on their clinical experience treating 538 patients with facial paralysis using pectoralis minor flaps. Their two-stage procedure involved dividing the pectoralis minor muscle tendon attachment into three parts, fixing them at the alar base, upper lip, and lower lip. The muscle was positioned over the zygomatic bone to mimic the zygomaticus muscle and elevate the upper lip. They found the fan-shaped form and robust tendon insertion of the pectoralis minor ideal for perioral transplantation.

In 2013, Dr. Alam and colleagues [53] first proposed the preclinical evaluation of using the sternohyoid muscle as a microvascular free flap for facial paralysis reconstruction, with the first clinical case published in 2016 [54]. The sternalis-myoid muscle originates from the posterior edge of the inner end of the clavicle, the posterior part of the costoclavicular ligament, and the upper and posterior parts of the manubrium. Arterial supply comes from the superior thyroid artery or lingual artery, venous drainage is provided by the corresponding veins, and the motor nerve is the descending branch of the sublingual nerve [55]. Compared to the gracilis muscle, the sternohyoid flap is similar in length, volume, and proportion of fast-twitch muscle fibers to the zygomaticus major. Additionally, the shorter distance between the origin and insertion points allows for a superior length-to-contraction ratio, enhancing facial symmetry during muscle contraction. However, a drawback of the sternohyoid muscle flap is the different positioning of its vascular pedicle and nerve pedicle, with the vascular pedicle at the flap’s head and the motor nerve at its tail. This discrepancy can lead to loose positioning of the flap at the optimal length, reducing muscle contraction efficiency [56].

### 2.3. Combined Multi-Flap Surgery 

A natural smile is generated by the contraction of several facial expression muscles, including the zygomaticus major, levator labii superioris, and levator palpebrae superioris [57]. Traditional dynamic reconstruction techniques have used a single muscle flap, such as the latissimus dorsi or gracilis muscle, to replace only one vector direction of the facial expression muscles. As a result, these techniques can only provide an unnatural restoration of facial expressions, with inadequate muscle contraction being a major problem in facial paralysis reconstruction. To address these shortcomings, Ueda et al. [58] reported a technique involving dual muscle transplantation to restore smiles. This method uses an LD flap innervated by the contralateral FN and a SAFF innervated by the ipsilateral hypoglossal nerve, replacing the zygomaticus, levator labii superioris, and depressor anguli oris muscles. This approach enables synchronized movement of both muscle flaps upon contralateral FN activation, producing a coordinated spontaneous smile. However, since only the ipsilateral hypoglossal nerve is used to innervate the SAFF, prolonged rehabilitation may be required to achieve coordinated movement of both flaps. In 2019, Hajime Matsumine et al. [41] introduced a single-stage dual muscle flap reconstruction technique using the contralateral FN and ipsilateral MN to innervate the LD and SAFF flaps. By connecting donor nerves to two different motor sources, both muscle flaps receive dual nerve innervation simultaneously. Multi-muscle flap surgery better mimics the direction of facial muscle movement, although it is more complex due to the harvesting of two muscle flaps and the need for multiple neurovascular anastomoses.

## 3. Discussion

Among various reconstruction options, vascularized nerve pedicle-free muscle flap transplantation is considered the gold standard for treating late-stage facial paralysis. The primary differences between techniques lie in the choice of donor muscle, motor nerve source, and whether a single-stage or multi-stage surgical approach is used.

Common motor nerve sources for muscle transfer include the ipsilateral MN and the contralateral FN via CFNG. CFNG effectively restores facial symmetry. Spontaneous smiling is a critical indicator of successful facial paralysis recovery. Research by Dr. Chuang and colleagues [59] suggests that the contralateral FN is the only neural regulator that can restore spontaneous smiles. However, the masseter nerve can also result in a spontaneous smile due to its high axon load and potential for cerebral adaptation [60,61,62,63]. Liang and other physicians opine [64] that both surgical procedures engendered a highly considerable primer excursion of the reanimated side and postoperative amelioration of the static or dynamic lip angle. Masseteric nerve coaptation elicited greater smile excursion and more marked improvement of the dynamic lip angle than CFNG. Dual nerve innervation has a positive impact on the generation of spontaneous smiles compared to single nerve innervation. This can be explained by the concept of neural boosting, where the masseter nerve enhances the neural signal, and the cross-facial nerve branch provides spontaneity. Clearly, one-stage surgery offers advantages, such as faster recovery time and alleviating the patient’s financial burden. Furthermore, due to the nerve action at the muscle’s distal attachment, potential nerve regeneration may be promoted [65]. Single-stage facial reconstruction is recommended for children with bilateral facial paralysis and adults with unilateral facial paralysis. Two-stage surgeries yield the best results in younger and thinner patients, possibly due to improved nerve regeneration and lighter muscle loads. The use of the serratus anterior and multi-muscle flaps better mimics the vectors of facial muscle movement, although this increases surgical complexity and requires advanced microsurgical skills. Consequently, single muscle flaps, such as the gracilis and LD flaps, remain the mainstream methods. Each surgical technique has its strengths and limitations. For instance, the masseter muscle flap offers high success rates but can result in horizontal tension and jaw contour issues. The temporalis muscle flap, while effective, may cause temporal concavity. The gracilis muscle flap, with dual nerve innervation, shows high success in spontaneous smiles but requires a longer recovery. The latissimus dorsi flap, despite its effectiveness, can lead to edema and shoulder issues. The serratus anterior free flap, though flexible, may not achieve adequate lip elevation (Table 1). The reviewed studies vary in their methodologies, making direct comparisons challenging. Additionally, long-term outcomes and patient-reported satisfaction are underreported, highlighting the need for standardized outcome measures in future research. Future research should focus on long-term outcomes, patient satisfaction, and standardizing surgical protocols to optimize treatment strategies.

## 4. Conclusions

Therefore, in the treatment of late-stage facial paralysis, a comprehensive evaluation of the patient by the physician is necessary. Considering the patient’s preferences, the most suitable surgical method should be chosen to maximize the restoration of the patient’s appearance and function.

## Figures and Tables

**Table 1:** Comparison of various surgical techniques for late-stage facial paralysis.

Technique	Advantages	Disadvantages	Spontaneous Movement Occurred Rate	Spontaneous Movement Occurred Time
Masseter Muscle Flap	High success rate; adjacent transfer without significant necrosis or denervation	Horizontal tension; jaw contour issues; lower excursion of the commissure upon smiling	/	/
Temporalis Muscle Flap	Proximity to surgical site; no need for nerve coaptation	Temporal concavity; swelling over zygomatic arch; possible eye closure during chewing	/	/
Gracilis Muscle Flap	Easy access for harvesting; minimal donor site impact; high success in spontaneous smiles	Need to lose a nerve across the face transplant; longer recovery	85–95%	5–6 months
Latissimus Dorsi Flap	Stable and rich blood supply; concealed donor site	Potential facial edema due to thickness; shoulder and back pain affecting mobility	85–90%	7–9 months
Serratus Anterior Free Flap	Flexibility in selecting skin paddle; ability to carry multiple muscle bundles	Inadequate lip elevation or cheek swelling; proximity of lip muscles may not achieve sufficient vector difference	75–85%	5–6 months

## Data Availability

All data can be provided from the correspondence author upon a reasonable request.
